# Laparoscopic Versus Open Cholecystectomy in Gangrenous Cholecystitis: A Retrospective Comparative Study

**DOI:** 10.7759/cureus.104682

**Published:** 2026-03-04

**Authors:** Ganesh Vadthya, Srikanth N Jarupla, Ajit K Naik, Rahul Tiwari, Anil Managutti, Seema Gupta

**Affiliations:** 1 Department of General Surgery, Deccan College of Medical Sciences, Hyderabad, IND; 2 Department of General Surgery, Dr BS Kushwah Institute of Medical Sciences, Kanpur, IND; 3 Department of General Surgery, Pandit Raghunath Murmu Medical College and Hospital, Baripada, IND; 4 Department of Oral and Maxillofacial Surgery, Narsinhbhai Patel Dental College and Hospital, Visnagar, IND; 5 Department of Orthodontics, Kothiwal Dental College and Research Centre, Moradabad, IND

**Keywords:** cholecystitis, complication, gangrenous, laparoscopic, open

## Abstract

Introduction: Gangrenous cholecystitis (GC) is a severe form of acute cholecystitis associated with increased operative difficulty and postoperative morbidity. Although open cholecystectomy is traditionally preferred, advances in minimally invasive surgery have expanded the role of laparoscopy. However, the optimal surgical approach to GC remains controversial. This study aimed to compare the perioperative and postoperative outcomes of laparoscopic and open cholecystectomy in patients with GC.

Materials and methods: This retrospective observational study was conducted at the Department of General Surgery at Deccan College of Medical Sciences, a tertiary care cente in Hyderabad, India. It included adult patients with intraoperatively confirmed GC who underwent laparoscopic or open cholecystectomy at a tertiary care center over a two-year period. Demographic data, operative parameters, intraoperative complications, postoperative morbidity, and length of hospital stay were analyzed and compared between groups.

Results: Seventy-nine patients were included, of which 34 underwent laparoscopic cholecystectomy and 45 underwent open cholecystectomy. Baseline demographic and clinical characteristics were comparable between the groups. The mean operative time was significantly longer in the laparoscopic group (98.5 ± 25.4 min) than in the open group (86.2 ± 22.1 min; p = 0.023). The intraoperative complication rates were similar (p = 0.87). Conversion to open surgery occurred in eight (23.5%) laparoscopic cases. The total number of postoperative complications showed a non-significant reduction following laparoscopic cholecystectomy (p = 0.052), not meeting the predefined threshold for statistical significance (p < 0.05). The median hospital stay was shorter in the laparoscopic group (6 vs. 8 days, p = 0.039).

Conclusion: Laparoscopic cholecystectomy is a safe and feasible option for GC, offering shorter hospital stay despite a longer operative time. A laparoscopic-first approach, with a low threshold for conversion, is recommended for appropriately selected patients.

## Introduction

Gangrenous cholecystitis (GC) represents the most severe form of acute cholecystitis and is associated with significant morbidity and mortality. It results from sustained gallbladder ischemia secondary to cystic artery compromise, leading to transmural necrosis, perforation, and severe local inflammation [1]. This condition is more commonly observed in elderly patients, males, and individuals with diabetes mellitus (DM) or cardiovascular comorbidities. Owing to its aggressive clinical course and frequent diagnostic delays, GC continues to pose major therapeutic challenges to surgeons [1,2].

Cholecystectomy is the definitive treatment for GC. Traditionally, open cholecystectomy was considered the procedure of choice because of dense adhesions, distorted anatomy, and perceived risk of bile duct injury during laparoscopic dissection [3]. However, with advancements in laparoscopic techniques, improved imaging, and growing surgical expertise, laparoscopic cholecystectomy has been increasingly attempted even in complicated gallbladder diseases [4]. Despite this shift, the optimal surgical approach to GC remains controversial.

Conflicting outcomes regarding operative time, conversion rates, postoperative complications, length of hospital stay, and overall recovery when comparing laparoscopic and open cholecystectomy in GC have been reported [5]. While laparoscopy offers the advantages of reduced postoperative pain, shorter hospitalization, and faster return to normal activity, concerns persist regarding higher conversion rates and intraoperative complications in the presence of severe inflammation and tissue friability [6]. Conversely, open cholecystectomy provides better tactile feedback and exposure but is associated with increased postoperative morbidity and prolonged recovery [7,8].

Given the lack of consensus, this study aimed to contribute additional data by analyzing the perioperative and postoperative outcomes associated with both surgical approaches. This study aimed to compare laparoscopic and open cholecystectomy in the management of GC. The objectives of this study were to compare perioperative and postoperative outcomes between laparoscopic and open cholecystectomy in patients with GC; to assess operative parameters, including operative time, intraoperative complications, and need for conversion; to evaluate postoperative morbidity such as surgical site infection, bile leak, and length of hospital stay; and to determine the safety and feasibility of laparoscopic cholecystectomy in the management of GC.

## Materials and methods

Study design and setting

This retrospective, non-randomized, observational comparative study was conducted in the Department of General Surgery at Deccan College of Medical Sciences, a tertiary care center in Hyderabad, India, over a period of two years, from August 2021 to August 2023. The medical records of patients who underwent laparoscopic or open cholecystectomy for GC were reviewed and analyzed. The choice of surgical approach was based on the surgeon’s discretion, intraoperative findings, and the patient’s clinical status. Ethical approval was obtained from the Institutional Ethics Committee (2024/69/051), and a waiver of informed consent was granted owing to the retrospective nature of the study. Patient confidentiality was strictly maintained, and all data were anonymized prior to analysis.

Sample size

As this was a retrospective observational study, formal sample size calculation was not performed. All consecutive patients who met the inclusion criteria and underwent laparoscopic or open cholecystectomy for GC during the study period were included in this analysis. Therefore, the sample size was determined based on the number of eligible cases available in the medical records over the defined study duration.

Study population

All patients diagnosed with GC and treated surgically during the study period were identified from the hospital medical records and assessed for eligibility. Patients were categorized into two groups based on the surgical procedure performed: laparoscopic cholecystectomy and open cholecystectomy. Cases in which laparoscopic cholecystectomy was converted to open surgery were analyzed according to the final surgical approach or reported separately as appropriate.

Inclusion and exclusion criteria

Patients aged ≥18 years with intraoperatively confirmed GC were included in this study. GC was defined by intraoperative findings, such as necrotic or friable gallbladder wall, patchy or diffuse gangrene, empyema, or perforation. Patients who underwent cholecystectomy for non-gangrenous acute or chronic cholecystitis, those managed conservatively, and those with incomplete clinical or operative records were excluded from the study.

Preoperative assessment

Preoperative clinical data were retrieved from patient records and included documented history, physical examination findings, and laboratory investigations, including complete blood count and liver function tests. Radiological evaluation consisted of ultrasonography in all patients, with computed tomography of the abdomen performed selectively where clinically indicated. Preoperative suspicion of GC was based on clinical severity and imaging findings; however, a definitive diagnosis was established intraoperatively.

Surgical technique

Details of the surgical procedure were obtained from the operative notes. Laparoscopic cholecystectomy was performed using the standard four-port technique. Adhesiolysis and dissection were performed carefully, with conversion to open surgery performed in cases of unclear anatomy, uncontrolled bleeding, or suspected bile duct injury. Open cholecystectomy was performed through a right subcostal incision, providing direct exposure of the gallbladder and Calot’s triangle [3]. Intraoperative findings and drain placement were documented at the surgeon’s discretion.

Outcome measures

The primary outcome measures included the operative time and intraoperative complications. Secondary outcome measures included postoperative complications, such as surgical site infection, bile leak, postoperative fever, and duration of hospital stay. Mortality, if any, was recorded. Data were obtained from the operative notes, postoperative progress charts, and discharge summaries.

Data collection

Clinical, operative, and postoperative variables were retrospectively collected from patients’ medical records using a structured data collection protocol. Data regarding demographics, comorbidities, intraoperative findings, and postoperative outcomes were systematically recorded to ensure consistency and completeness.

Statistical analysis

Data were entered into a spreadsheet and analyzed using the appropriate statistical software (IBM SPSS Statistics for Windows, Version 22.0, released 2013, IBM Corp., Armonk, NY). Normality of continuous variables was assessed using the Shapiro-Wilk test. Normally distributed variables were expressed as mean ± standard deviation (SD) and compared using the independent samples t-test. Non-normally distributed variables were expressed as median and interquartile range (IQR) and compared using the Mann-Whitney U test. Categorical variables were expressed as frequencies and percentages. Between-group comparisons were performed using the Chi-square test. When expected cell counts were <5, Fisher’s exact test was applied. A two-tailed p-value <0.05 was considered statistically significant. Patients in whom laparoscopic cholecystectomy was initially attempted but required intraoperative conversion to open surgery were analyzed within the laparoscopic group according to the initial surgical intent (intention-to-treat principle). This approach was adopted to avoid post hoc reclassification and potential analytical bias.

## Results

No mortality was observed in either group during the study period. The two surgical cohorts were well-matched at baseline. Inflammatory markers and liver enzymes were also comparable between the groups. The absence of significant demographic or clinical differences established that the laparoscopic and open groups were equivalent in disease severity and patient profile at presentation. This baseline comparability is crucial, as it strengthens the internal validity of the study and allows for a more confident attribution of any observed differences in operative or postoperative outcomes to the surgical approach itself, rather than to confounding preexisting factors (Table 1).

**Table 1:** Baseline demographic, clinical, and laboratory characteristics of patients with gangrenous cholecystitis according to surgical approach. Data are presented as mean ± standard deviation, median (interquartile range), or number (percentage), as appropriate. No formal statistical comparisons were performed for baseline variables. BMI: body mass index; CRP: C-reactive protein; ALP: alkaline phosphatase; AST: aspartate aminotransferase

Variables		Laparoscopic cholecystectomy (n = 34)	Open cholecystectomy (n = 45)
Age (years)	Mean ± SD	52.5 ± 19.5	56.7 ± 21.4
BMI (kg/m²)	Mean ± SD	25.4 ± 6.5	26.3 ± 12.5
Duration of symptoms (days)	Mean ± SD	3.1 ± 2.8	4.6 ± 5.8
Preoperative diffuse peritonitis	n (%)	1 (2.9%)	1 (2.2%)
Thickened gallbladder wall	n (%)	22 (64.7%)	24 (53.3%)
C-reactive protein (mg/L)	Median (IQR)	136 (88)	124 (83)
Leukocyte count (10⁹/L)	Median (IQR)	9.03 (3.58)	11.3 (8.31)
ALP (U/L)	Mean ± SD	178 ± 45	185 ± 52
AST (U/L)	Mean ± SD	88 ± 22	82 ± 30
Serum lipase (U/L)	Mean ± SD	65 ± 40	72 ± 55

The mean operating time was significantly longer for laparoscopic cholecystectomy (98.5 min vs. 86.2 min, p = 0.023). However, the rates of intraoperative complications were statistically similar between the groups (p = 0.87), including specific events, such as gallbladder perforation (p = 0.702) and significant bleeding (p = 0.692). Conversion to open surgery was required in 8 (23.5%) laparoscopic attempts. The laparoscopic approach for GC is technically more demanding, as evidenced by the longer operative time and higher conversion rate. However, this increased complexity did not translate into a higher incidence of immediate intraoperative complications, demonstrating that the procedure can be performed with intraoperative safety comparable to that of open surgery when performed by experienced surgeons, albeit with a need for contingency planning for conversion (Table 2).

**Table 2:** Intraoperative outcomes and operative parameters in laparoscopic and open cholecystectomy groups. Data are presented as mean (standard deviation) or number (percentage). The mean operating time was compared using the independent samples t-test, Categorical variables were analyzed using the †Chi-square test or ††Fisher’s exact test, as appropriate. *Statistically significant at p < 0.05, N/A: not applicable.

Outcome	Laparoscopic cholecystectomy (n = 34)	Open cholecystectomy (n = 45)	Test statistics	p-value
Mean operating time (min), Mean ± SD	98.5 ± 25.4	86.2 ± 22.1	2.33	0.023*
Intraoperative complications, n (%)	10 (29.4%)	14 (31.1%)	0.03	0.870†
- Gallbladder perforation	8 (23.5%)	9 (20.0%)	0.15	0.702†
- Significant bleeding (>100 mL)	2 (5.9%)	5 (11.1%)	-	0.692††
Conversion to open surgery, n (%)	8 (23.5%)	N/A	N/A	N/A

Patients who underwent laparoscopic surgery experienced a significantly lower total postoperative complication rate (p = 0.05) and shorter median hospital stay (six days vs. eight days, p = 0.039). Although not individually significant, the rates of surgical site infection (p = 0.066) and major complications (p = 0.178) were numerically lower in the laparoscopic group. Successfully completed laparoscopic cholecystectomy for GC is associated with a superior postoperative recovery profile compared with open surgery. The significant reduction in overall morbidity and shorter hospitalization highlights a clear clinical benefit, suggesting that the laparoscopic approach, when feasible, enhances patient recovery and optimizes healthcare resource utilization despite intraoperative technical challenges (Table 3).

**Table 3:** Postoperative morbidity and length of hospital stay following laparoscopic and open cholecystectomy. Data are presented as number (percentage) or median (interquartile range), Hospital stay was compared using the Mann–Whitney U test, Categorical outcomes were analyzed using the †Chi-square test or ††Fisher’s exact test, as appropriate. *Statistically significant at p < 0.05.

Complication/outcome	Laparoscopic cholecystectomy (n = 34)	Open cholecystectomy ( n= 45)	Test statistic	p-value
Total complications, n (%)	8 (23.5%)	20 (44.4%)	3.84	0.052†
Major complications, n (%)	2 (5.9%)	8 (17.8%)	-	0.178††
- Surgical site infection	3 (8.8%)	11 (24.4%)	3.39	0.066†
- Bile leak	1 (2.9%)	4 (8.9%)	-	0.38††
- Reoperation	1 (2.9%)	3 (6.7%)	-	0.628††
Minor complications, n (%)	6 (17.6%)	12 (26.7%)	0.92	0.338†
Hospital stay (days), median (IQR)	6 (3-9)	8 (5-10)	U = 558.5	0.039*

## Discussion

GC represents the most severe end of the spectrum of acute cholecystitis and is associated with increased operative difficulty, postoperative morbidity, and mortality compared with uncomplicated diseases [1,9]. Traditionally, open cholecystectomy was advocated as the preferred surgical approach because of dense adhesions, friable gallbladder tissue, and distorted biliary anatomy, which are thought to increase the risk of bile duct injury during laparoscopic dissection [3]. However, advances in laparoscopic instrumentation, improved imaging, and growing surgeon experience have challenged this paradigm, prompting the reevaluation of the role of laparoscopy, even in complicated gallbladder diseases [4].

In the present study, laparoscopic cholecystectomy was associated with a significantly longer operative time than open cholecystectomy. This finding is consistent with a previous study reporting an increased operative duration during laparoscopic management of symptomatic retained gallstones following previous open partial or incomplete cholecystectomy [10]. Chang et al. [11] reported that prolonged operative time reflects technical complexity rather than procedural inefficiency, and should not be considered a negative outcome in isolation. Importantly, despite longer operative times, intraoperative complication rates in our study were comparable between the laparoscopic and open approaches, supporting the intraoperative safety of laparoscopy when performed by experienced surgeons.

The conversion rate observed in this study was comparable to previously reported rates ranging from 15% to 35% in patients with GC [12]. Conversion should be viewed as a strategic decision aimed at preventing serious complications, rather than as a failure of the laparoscopic approach. In the present study, no bile duct injuries were observed in either group. Conversion to open surgery should therefore be viewed as a safety strategy rather than a procedural failure. External evidence suggests that timely conversion in difficult cases may reduce the risk of major biliary injury [13]; however, our findings neither confirm nor refute this association due to the absence of such events in our cohort. A previous study reported that increased conversion was seen in adults > 65 years of age, obesity, surgeries performed by senior surgeons, and patients with ultrasonographic findings of a thickened gallbladder wall [14].

One of the most clinically relevant findings of this study was the significantly lower total postoperative complication rate in the laparoscopy group. Although individual complications such as surgical site infection and bile leak did not reach statistical significance, they were consistently less frequent after laparoscopic cholecystectomy. These trends are in agreement with those of multiple studies demonstrating reduced wound-related complications and postoperative infections with minimally invasive surgery [13,15]. In contrast, open cholecystectomy has been associated with higher rates of wound infection, postoperative pain, pulmonary complications, and delayed recovery, particularly in elderly patients and those with comorbidities who are disproportionately affected by GC [16].

The significantly shorter hospital stay in the laparoscopic group further highlights the postoperative advantages of minimally invasive surgery. Reduced length of stay following laparoscopic cholecystectomy has been consistently reported in the literature and is attributed to less postoperative pain, earlier mobilization, faster return of bowel function, and lower wound morbidity [15-17]. From a healthcare system perspective, shorter hospitalization translates into reduced costs and improved resource utilization, which is particularly relevant in high-volume tertiary care centers.

An important strength of this study was the baseline comparability between the laparoscopic and open groups with respect to demographic characteristics, inflammatory markers, and biochemical parameters. This suggests that the disease severity at presentation was similar between the groups and reduced the likelihood that postoperative differences were driven by preoperative confounding factors. Although measured baseline demographic and laboratory variables were comparable between groups, the retrospective and non-randomized design limits causal inference. Unmeasured confounders, including surgeon preference, perceived operative difficulty, and subtle differences in disease severity, may have influenced treatment allocation and outcomes. Therefore, differences observed between groups cannot be attributed solely to the surgical approach.

Overall, the findings of this study align with the growing evidence that laparoscopic cholecystectomy is both feasible and beneficial in selected patients with GC. Although technical difficulty, longer operative time, and conversion remain important considerations, these factors are outweighed by reduced postoperative morbidity and shorter hospital stay. Open cholecystectomy continues to play a critical role in cases of severe inflammation, unclear anatomy, or hemodynamic instability; however, it should no longer be regarded as the default approach for all patients with GC.

Clinical implications

When performed by experienced surgeons, laparoscopic cholecystectomy can be safely adopted as the initial surgical approach for GC in stable patients. Therefore, a low threshold for conversion to open surgery should be maintained to ensure patient safety. The observed reduction in postoperative complications and hospital stays supports a laparoscopic-first strategy, which may improve patient recovery and optimize healthcare resource utilization.

Limitations

A major limitation of this study is the non-randomized allocation of the surgical approach. The decision to perform a laparoscopic or open cholecystectomy was based on the surgeon's discretion, intraoperative judgment, and patient condition. This introduces a substantial risk of selection bias. Surgeons may have preferentially selected laparoscopic surgery for clinically stable patients or those perceived to have less severe inflammation, while reserving open surgery for more complex or higher-risk cases. Although baseline demographic and laboratory parameters were comparable between groups, unmeasured factors such as anatomical distortion, degree of local inflammation, hemodynamic stability, and surgeon experience were not controlled for and may have influenced both treatment allocation and outcomes. Therefore, residual confounding cannot be excluded, and causal inferences should be interpreted with caution. The single-center nature limits the generalizability of our results. The relatively small sample size and absence of a priori power calculation limit the statistical power of the study, particularly for detecting differences in infrequent complications. Therefore, non-significant findings should not be interpreted as evidence of equivalence between surgical approaches, and type II error cannot be excluded. Long-term outcomes, such as incisional hernia, quality of life, and cost-effectiveness, were not evaluated and should be addressed in future prospective multicenter studies.

## Conclusions

Laparoscopic cholecystectomy is a safe and feasible option for the management of GC in appropriately selected patients. Although associated with longer operative time and a notable conversion rate, it offers reduced postoperative morbidity and shorter hospital stay compared with open cholecystectomy. In this tertiary care setting, procedures were performed or supervised by experienced consultant surgeons, which is an important factor in ensuring operative safety. Conversion to open surgery should be considered a prudent intraoperative decision when anatomical distortion or technical difficulty compromises safety. These findings support a laparoscopic-first approach in hemodynamically stable patients when performed in centers with adequate surgical expertise.
